# A hybridization target enrichment approach for pathogen genomics

**DOI:** 10.1128/mbio.01889-23

**Published:** 2023-10-13

**Authors:** Balaji Sundararaman, Matthew D. Sylvester, Varvara K. Kozyreva, Zenda L. Berrada, Russell B. Corbett-Detig, Richard E. Green

**Affiliations:** 1 Department of Biomolecular Engineering, University of California Santa Cruz, Santa Cruz, California, USA; 2 Center for Laboratory Sciences, California Department of Public Health, Microbial Diseases Laboratory Branch, Richmond, California, USA; 3 UCSC Genomics Institute, University of California Santa Cruz, Santa Cruz, California, USA; Northern Arizona University, Flagstaff, Arizona, USA

**Keywords:** genomics, genomic epidemiology, antibiotic resistance, whole-genome enrichment

## Abstract

Genomic epidemiology uses pathogens’ whole-genome sequences to understand and manage the spread of infectious diseases. Whole-genome data can be used to monitor outbreaks and cluster formation, identify cross-community transmissions, and characterize drug resistance and immune evasion. Typically, bacteria are cultured from clinical samples to obtain DNA for sequencing to generate whole-genome data. However, culture-independent diagnostic methods are utilized for some fastidious bacteria for better diagnostic yield and rapid pathogen genomics. Whole-genome enrichment (WGE) using targeted DNA sequencing enables direct sequencing of clinical samples without having to culture pathogens. However, the cost of capture probes (“baits”) limits the utility of this method for large-scale genomic epidemiology. We developed a cost-effective method named *C*ircular *N*ucleic acid *E*nrichment *R*eagent *s*ynthesis (CNERs) to generate whole-genome enrichment probes. We demonstrated the method by producing probes for *Mycobacterium tuberculosis*, which we used to enrich *M. tuberculosis* DNA that had been spiked at concentrations as low as 0.01% and 100 genome copies against a human DNA background to 1,225-fold and 4,636-fold. Furthermore, we enriched DNA from different *M. tuberculosis* lineages and *M. bovis* and demonstrated the utility of the WGE-CNERs data for lineage identification and drug-resistance characterization using an established pipeline. The CNERs method for whole-genome enrichment will be a valuable tool for the genomic epidemiology of emerging and difficult-to-grow pathogens.

**IMPORTANCE:**

Emerging infectious diseases require continuous pathogen monitoring. Rapid clinical diagnosis by nucleic acid amplification is limited to a small number of targets and may miss target detection due to new mutations in clinical isolates. Whole-genome sequencing (WGS) identifies genome-wide variations that may be used to determine a pathogen’s drug resistance patterns and phylogenetically characterize isolates to track disease origin and transmission. WGS is typically performed using DNA isolated from cultured clinical isolates. Culturing clinical specimens increases turn-around time and may not be possible for fastidious bacteria. To overcome some of these limitations, direct sequencing of clinical specimens has been attempted using expensive capture probes to enrich the entire genomes of target pathogens. We present a method to produce a cost-effective, time-efficient, and large-scale synthesis of probes for whole-genome enrichment. We envision that our method can be used for direct clinical sequencing of a wide range of microbial pathogens for genomic epidemiology.

## INTRODUCTION

Genomic epidemiology uses whole-genome sequencing (WGS) data to understand and manage infectious diseases (1, 2). Genomes of several pathogens have been used to study small outbreaks (3) and to track strain prevalence at national and global levels (1, 2). Genomic data have been used to identify the origin and introduction of new strains, identify outbreaks, and reveal how clinical isolates are related in space and time (4, 5). WGS has been used to identify drug-resistance bacterial outbreaks (6) and to genotype and track transmission dynamics (4). WGS data are used to predict pathogen serotypes (7) and phenotypes, including virulence factors (8), drug resistance (6, 9, 10), and immune evasion factors (11). WGS data are also used to characterize phylogenetic relationships at a higher resolution than multi-locus markers, allowing the identification of fine-scale relationships between pathogenic strains in national (5, 12) and global surveillance programs (13).

The 2019 severe acute respiratory syndrome coronavirus-2 (SARS-CoV-2) pandemic demonstrated the potential of genomic epidemiology in real-time disease monitoring. Since 2019, >15 million SARS-CoV-2 genomes have been sequenced (https://gisaid.org/) to understand transmission dynamics and track viral evolution in response to clinical interventions (14). The SARS-CoV-2 pandemic also advanced the technologies and computational capabilities needed to produce and analyze big data for genomic epidemiology (15). Motivated by the success of SARS-CoV-2 genomic surveillance, there is renewed interest in applying genomic epidemiology to monitor other emerging pathogens, predict outbreaks, and recommend control measures.

Genomic data are generated by WGS for pathogens. WGS requires high quality and a reasonable quantity of pathogen DNA, which is often difficult to obtain from clinical samples (16). Bacterial pathogens are generally grown *in vitro* to produce DNA for sequencing (17). Culturing pathogens for DNA is routinely done for bacterial pathogens that are simple to grow (18). However, culturing poses a hurdle for some fastidious bacterial and fungal pathogens. These difficult-to-culture pathogens are usually slow-growing or require special growth conditions such as the presence of host cells, nutrient-rich media, and special environments (18, 19). Furthermore, culturing steps can alter the diversity that was initially present in clinical samples (20, 21). Culture-independent direct sequencing of clinical samples can overcome many of these challenges (22, 23).

Metagenomic methods have been developed to directly sequence clinical samples such as sputum, cerebrospinal fluid, and urine to detect and assemble pathogen genomes in an unbiased fashion (24 – 27). However, the presence of high amounts of human DNA and commensal microbiome DNA can obscure the detection of trace amounts of pathogen DNA (24, 26). In addition, metagenomic methods often fail to achieve the high sequence coverage required for variant detection, which is needed for phenotypic and phylogenetic characterization. Depletion of human DNA and/or enrichment of pathogen DNA have been used in culture-independent diagnostic tests (22 – 27). Various sample processing methods have also been developed to lyse human cells to remove human DNA before lysing pathogens (23). However, these methods often fail to achieve the depth of sequencing coverage required for genomic epidemiology due to the presence of other microbial DNA.

Whole-genome enrichment (WGE) of bacterial DNA achieves higher genome coverage in direct sequencing of clinical samples (24). Multiplex polymerase chain reaction (PCR) using sequence-specific primers or cDNA amplification using random primers enable WGE for viral genomes due to their smaller size (27 – 29). Hybridization capture using biotinylated RNA or DNA probes, also known as baits, has been used to enrich large genomes of bacterial, fungal, and parasitic pathogens from clinical samples (19 – 22, 30, 31). However, large-scale synthesis of baits that cover entire genomes is often prohibitively expensive (24, 31). Current bait synthesis methods are inadequate to meet the large-scale requirements of genomic epidemiology.

We developed the *C*ircular *N*ucleic acid *E*nrichment *R*eagent *s*ynthesis (CNERs) method for cost-effective, large-scale DNA bait synthesis. The CNERs method can be used to make custom-designed probes to capture specific target regions in a genome, as we demonstrated previously (32). By substituting bioinformatically designed and chemically synthesized DNA oligonucleotides with genomic fragments of a target organism as templates (Fig. S1), the CNERs method can also be used to generate probes to enrich the entire genome of the target organism. The use of genomic DNA fragments as templates eliminates the need for chemically synthesized oligos, which reduces the cost of making CNERs significantly. Here, we describe this approach to make baits against an entire target pathogen genome and demonstrate the CNERs method to produce DNA baits for WGE of *Mycobacterium tuberculosis* (*M. tuberculosis*). We enriched *M. tuberculosis* DNA from an initial representation of 0.01% spiked with human DNA to a final representation of >85% using *M. tuberculosis* WGE-CNERs. We also captured a panel of various *M. tuberculosis* lineages and *M. bovis* [another species within *M. tuberculosis* complex (MTBC)] and several non-tuberculous mycobacteria (NTMs), demonstrating the sensitivity and specificity of WGE-CNERs. Furthermore, we showed the utility of the WGE data generated using CNERs for lineage identification and drug resistance characterization.

## RESULTS

We previously demonstrated the CNERs method to generate DNA baits against specific genomic target regions (32), which can be adopted to make probes against entire genomes. To generate WGE-CNERs, the genomic DNA (gDNA) of a target pathogen or related taxa is fragmented (Fig. S1) and circularized by splint ligation using a bridge adapter. The bridge adapter contains an upper oligo with a rare cutter restriction enzyme recognition site (RES) and oligo-dT sequences; the bottom oligo is complimentary to the upper oligo with degenerate nucleotides at both ends (Fig. S1). These degenerate nucleotides randomly complement the ends of target gDNA to facilitate splint ligation of the upper oligo. Ligation of the upper oligo both circularizes and incorporates RES and oligo-dT sequences in the target gDNA, regardless of their sequences (Fig. S1). Circularized templates are then amplified by rolling circle amplification (RCA) and digested as described in Sundararaman et al. (32) to generate double-stranded CNERs. WGE-CNERs can be used as baits to capture whole genomes of target species and related taxa.

For this study, we sheared ~286 ng of *M. tuberculosis* strain H37Rv gDNA (Fig. S2A) and generated gDNA fragments with a 143-bp average size (Fig. S2B). We circularized 100 ng sheared gDNA with a bridge adapter. RCA amplification of circularized templates yielded 4,760 ng of ~37 kB mean size high molecular weight DNA (Fig. S2C). HindIII restriction digestion of RCA products generated 4,640 ng of monomeric CNERs with an average size of 131 bp (Fig. S2D). In another experiment, we tested 50 ng sheared gDNA as an input template, generating 1.8 µg CNERs. Thus, we estimate that ~50 ng of sheared gDNA can produce ~2 µg WGE-CNERs.

### CNERs efficiently enriches the whole genomes of *M. tuberculosis*

We made four contrived mixtures of *M. tuberculosis* and human gDNA Next Generation Sequencing (NGS) libraries with unique Illumina dual indices. Sequencing before enrichment confirmed the expected *M. tuberculosis* read representation (Table 1). Sequencing after enrichment using *M. tuberculosis* WGE-CNERs yielded 84%–99% *M. tuberculosis* data, representing a 9.9–1,225.3-fold enrichment that varied based on initial proportions (Table 1). Correspondingly, the human libraries were 0.01–0.16-fold depleted to a final read representation of 1%–15.5%.

**TABLE 1:** WGE of *M. tuberculosis* from a contrived mixture with human libraries

Mixture	*M. tb* before capture (expected)	*M. tb* before capture (actual)	*M. tb* after capture	*M. tb* fold enrichment	Human before capture (expected)	Human before capture (actual)	Human after capture	Human fold depletion
1	10.000%	9.983%	98.974%	9.91	90.000%	90.017%	1.026%	0.01
2	1.000%	1.249%	98.731%	79.07	99.000%	98.751%	1.269%	0.01
3	0.100%	0.153%	91.415%	599.32	99.900%	99.847%	8.585%	0.09
4	0.010%	0.069%	84.450%	1,225.28	99.990%	99.931%	15.550%	0.16

**FIG 1:**
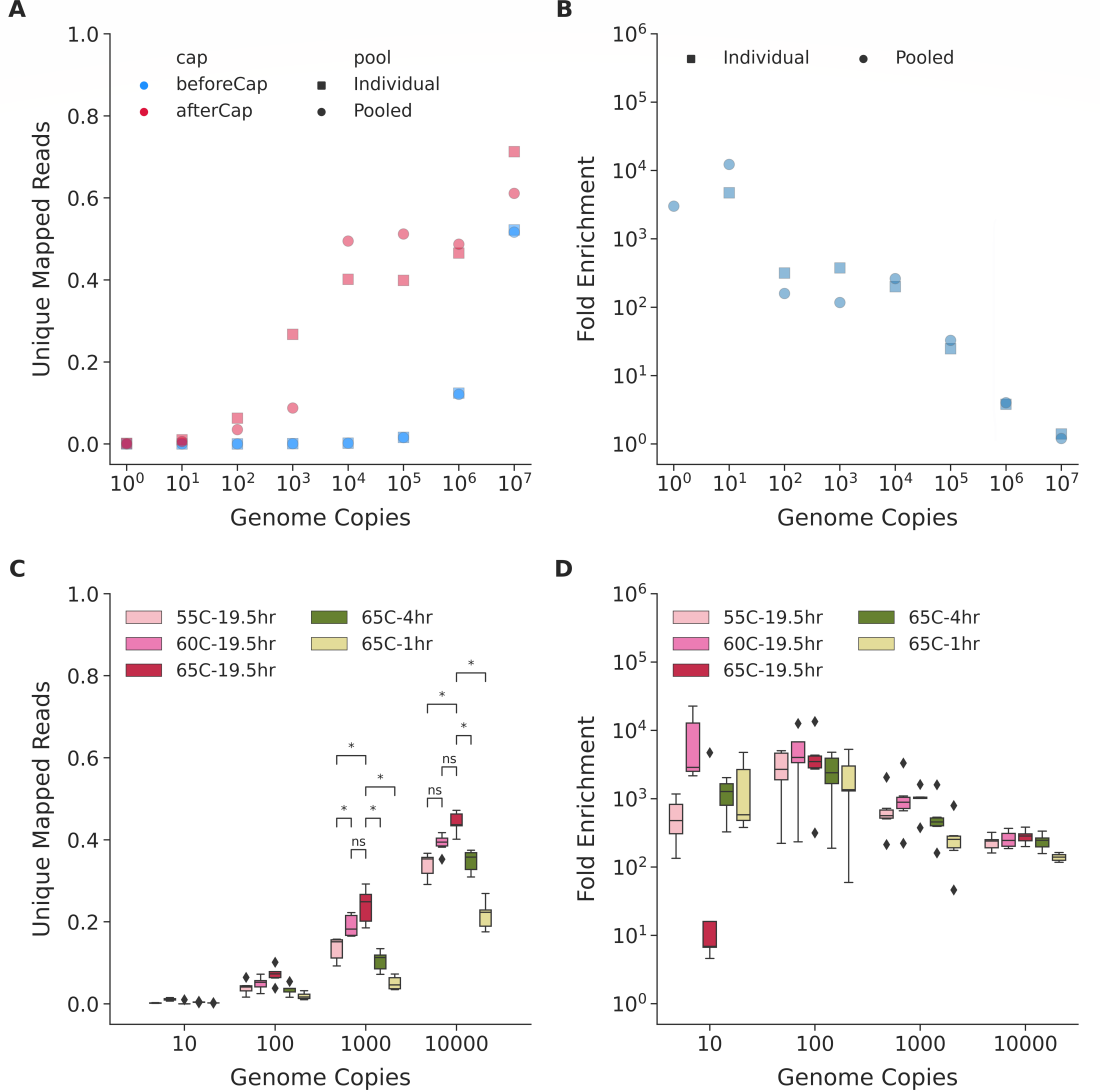
CNERs efficiently enriches MTBC DNA spiked with human DNA. (**A**) Percentage of unique mapped reads before (cyan) and after (pink) enrichment with CNERs for individual (squares) and pooled (dots) libraries with 1 × 10^1^–1 × 10^7^ *M. tuberculosis* genome copies. (**B**) Fold enrichment, which is the ratio between after and before enrichment unique mapped reads for individual and pooled capture experiments shown in panel A. (**C**) Box plots of the percentage of unique mapped reads after enrichment at the indicated hybridization temperatures and times for the five *M. tuberculosis* lineages and *M. bovis*. (**D**) Fold enrichment at the indicated hybridization temperatures and times. Asterisks denote statistical significance by the Mann-Whitney Wilcox test.

To test the lowest genome copies that *M. tuberculosis* WGE-CNERs can enrich, we spiked *M. tuberculosis-*H37Rv gDNA equivalent to 1 × 10^0^–1 × 10^7^ genome copies with human DNA. Sequencing before enrichment produced 0%–52% of reads uniquely mapping to the *M. tuberculosis-*H37Rv reference genome (NC_000962.3, Table S1) and is consistent between two PCR replicates (cyan squares and circles, Fig. 1A). Percent mapped reads exponentially increased, corresponding to the 10-fold increase in genome copies. We enriched the mixtures either individually for each copy number or pooled all the mixtures before enrichment. Percent unique mapped reads exponentially increased after enrichment from 0.1% to 17.7% for 1 × 10^0^–1 × 10^3^ copies and plateaued at ~45% for 1 × 10^4^–1 × 10^6^ copies and reached ~66% for 1 × 10^7^ copy mixture (pink squares and dots, Fig. 1A; Table S1). Percent unique mapped reads differed 2.5-fold between individual and pooled captures of the same copy mixtures for 1 × 10^0^–1 × 10^3^ copies and about 6% for 1 × 10^4^–1 × 10^7^ copy mixtures (Table S1).

We determined the fold enrichment as the ratio between before and after enrichment percent unique mapped reads (Fig. 1B). Fold enrichment for one-copy mixture is unreliable due to inconsistencies in percent mapped reads. Enrichment of a 10-copy mixture produced 4,717× fold enrichment for individual and 12,272× for pooled capture experiments. One hundred- and 1,000-copy mixtures produced 117×–375× (average 241×) fold enrichments that differed ~1.5-fold between individual and pooled captures. The fold enrichment exponentially decreased from 200× to 1.3× with increasing copies for 1 × 10^4^–1 × 10^7^ mixtures that differed ~10% between individual and pooled capture experiments of the same copy numbers (Table S1).

Pairwise comparison of normalized coverage of 100 bp bins across the genome shows that coverage after CNERs enrichment is highly correlated for samples with >10,000 copies (Pearson’s *r* = 0.90–0.93, Fig. S3). Normalized coverage shows that certain genomic regions are preferentially enriched at a given sequencing depth when ≤1,000 copies are present (Fig. S3). Eventually, the pairwise correlation for these samples decreases compared to higher-copy samples. The average Pearson’s *r* values are 0.87, 0.70, 0.37, and 0.095, respectively, for 1,000, 100, 10, and 1 copy samples compared to higher-copy samples.

### CNERs specifically enrich MTBC lineages and species

We asked whether CNERs made using the *M. tuberculosis*-H37Rv gDNA can enrich different species and lineages of MTBC and NTM. We made libraries for 1 × 10^1^–1 × 10^4^ genome copies of four *M. tuberculosis* lineages (Indo-Oceanic [L1] , East-Asian [L2], East-African-Indian [L3] , and Euro-American [L4]), *M. bovis*, and three NTM species (*Mycobacteroides abscessus*, *Mycobacterium fortuitum*, and *Mycobacterium porcinum*) mixed with human DNA. We generated ~153k read pairs for each library before enrichment, which resulted in 0%–0.2% unique mapped reads, which varied based on genome copies as expected (Table S1).

We pooled the same copy number libraries of different taxa and captured them at 55°C, 60°C, and 65°C to test the effect of hybridization temperature on capture efficiency. We sequenced ~53k read pairs for each library after enrichment, which generated 0%–47.5% unique mapped reads when mapped to the *M. tuberculosis*-H37Rv reference genome NC_000962.3 (shades of pink, Fig. 1C; Table S1). Similar to the H37Rv captures, unique mapped reads increased with increasing copy numbers for *M. tuberculosis* lineages and *M. bovis* (Fig. 1C) but did not improve mapped reads for NTMs (Fig. S4; Table S1). NTMs produced <7% unique mapped reads (Fig. S4). The low percentage of unique mapped reads for NTM samples might be due to poor capture of NTM DNA by *M. tuberculosis* WGE-CNERs or poor mapping of NTM reads to the NC_000962.3 reference. To test this, we mapped after-enrichment reads of NTMs to three NTM references (Fig. S4) that produced ~9.3% unique mapped reads on average. The mapped reads slightly differed between the three NTM references. Mapping to the individual NTM references showed that the low percent mapped reads are due to poor capture of NTM genomes by *M. tuberculosis* CNERs rather than poor mapping to *M. tuberculosis* reference. Five-fold differences between *M. tuberculosis* and NTMs in the after-enrichment unique mapped reads demonstrate that *M. tuberculosis* WGE-CNERs is specific to the MTBCs and does not enrich NTM genomes. The modest improvements in the percent mapped reads for NTMs between before and after enrichment and 1× read coverage of ~6% of *M. tuberculosis* reference genome in NTM samples (Table S1) indicate that *M. tuberculosis* WGE-CNERs might enrich small portions of conserved regions in the genomes of all mycobacterial species.

For MTBC, hybridization at 65°C produced on average 44.2% and 23.9% unique mapped reads, compared to 39.1% and 19.0% at 60°C and 33.8% and 13.5% at 55°C for the 10,000- and 1,000-copy mixtures, respectively. For the 10- and 100-copy mixtures, the percent unique mapped reads were <10% and did not significantly differ between different hybridization temperatures (Fig. 1C; Fig. S5; Table S1). The fold enrichment decreased with increasing copy numbers, similar to the H37Rv captures (Fig. 1D; Fig. S6; Table S1). WGE of the 10,000-copy mixture resulted in an average 250-fold enrichment.

For rapid clinical diagnostic applications, reducing overnight hybridization to ≤4 h would allow for a 1-day enrichment protocol. To explore this, we enriched the MTBC mixture for 1 or 4 h hybridizations at 65°C. Hybridization for 1 h produced on average 21.6% and 5.0% unique mapped reads compared to 34.8% and 10.5% for 4 h and 44.2% and 23.9% for 19.5 h at 65°C (data from the temperature experiments) for the 10,000- and 1,000-copy mixtures, respectively (green and yellow bars, Fig. 1C; Table S1). Hybridization duration does not significantly change the percent unique mapped reads for the 10- and 100-copy mixtures.

### CNERs enrichment produces high-coverage MTBC genomes

We deeply sequenced after-enrichment libraries for samples captured at 65°C for 19.5 h to analyze genomic coverage. To independently verify the origin of reads, we Blast searched raw reads against the NCBI nucleotide (nt) database (33) and analyzed the search results using Metagenomic Analyzer (MEGAN) software (34). MEGAN assigned 0.5%, 12.5%, 37.1% and 53.8% of reads to *M. tuberculosis* taxa on average for the five *M. tuberculosis* lineages and *M. bovis* samples for the 10-, 100-, 1,000- and 10,000-copy mixtures, respectively (Fig. S7). The percent of taxa assigned as *M. tuberculosis* is consistent with the percent mapped reads to the NC_000962.3  reference genome (Fig. 1C).

**FIG 2:**
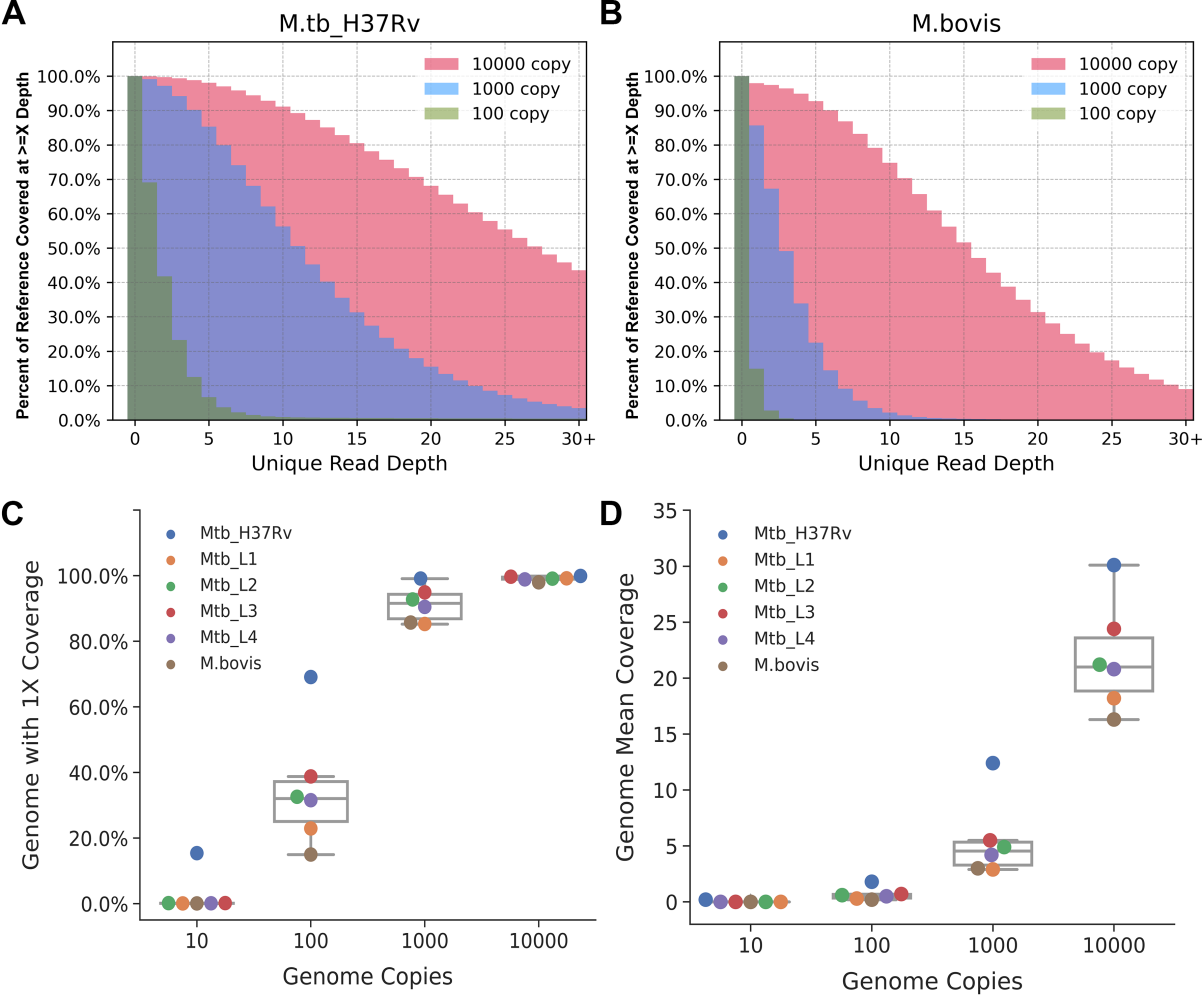
Breadth and depth of genome coverage using WGE-CNERs data. Overlapping histogram of percent of NC_000962.3 reference bases with X or more unique read depth from 3 million reads of WGE-CNERs data for 10,000-copy (pink), 1,000-copy (cyan), and 100-copy (green) mixtures of *M. tuberculosis*-H37Rv (**A**) and *M. bovis* (**B**). Box plots with overlapping swarm plots of percent of genome with 1× or more read coverage (**C**) and genome unique mean coverage (**D**) resulting from 3 million reads of WGE-CNERs data from five *M. tuberculosis* lineages and *M. bovis* (color dots) at the indicated copy numbers.

We subsampled 3 million raw reads corresponding to ~102× of *M. tuberculosis* genomic coverage that produced 0%, 0.7%, 7.1%, and 28.2% unique mapped reads with 81.2%, 93.7%, 82.1%, and 53.9% duplication rates for the four copy mixtures. We measured the coverage at each genomic position and plotted the percentage of the NC_000962.3 reference bases covered with X or more unique reads (Fig. 2). The coverage for *M. tuberculosis-*H37Rv (Fig. 2A) for a given copy mixture is slightly better than that for *M. bovis* (Fig. 2B) and other *M. tuberculosis* lineages (Fig. S8). We analyzed the breadth of coverage by looking at the NC_000962.3 reference bases covered with ≥1 read (1× coverage). For *M. tuberculosis*-H37Rv, the 1× coverage for the four copy mixtures is 15.4%, 69.1%, 99.1%, and 99.9% (blue dots, Fig. 2C) compared to 0%, 28.1%, 89.8%, and 99.0% on average for the other four lineages and *M. bovis* (Fig. 2C). At the given 3 million raw read pairs, the unique mean coverage depth for H37Rv is 0.2, 1.8, 12.4, and 30.1 (blue dots, Fig. 2D) compared to 0, 0.5, 4.1, and 20.2 for the other five MTBC samples (Fig. 2D).

**FIG 3:**
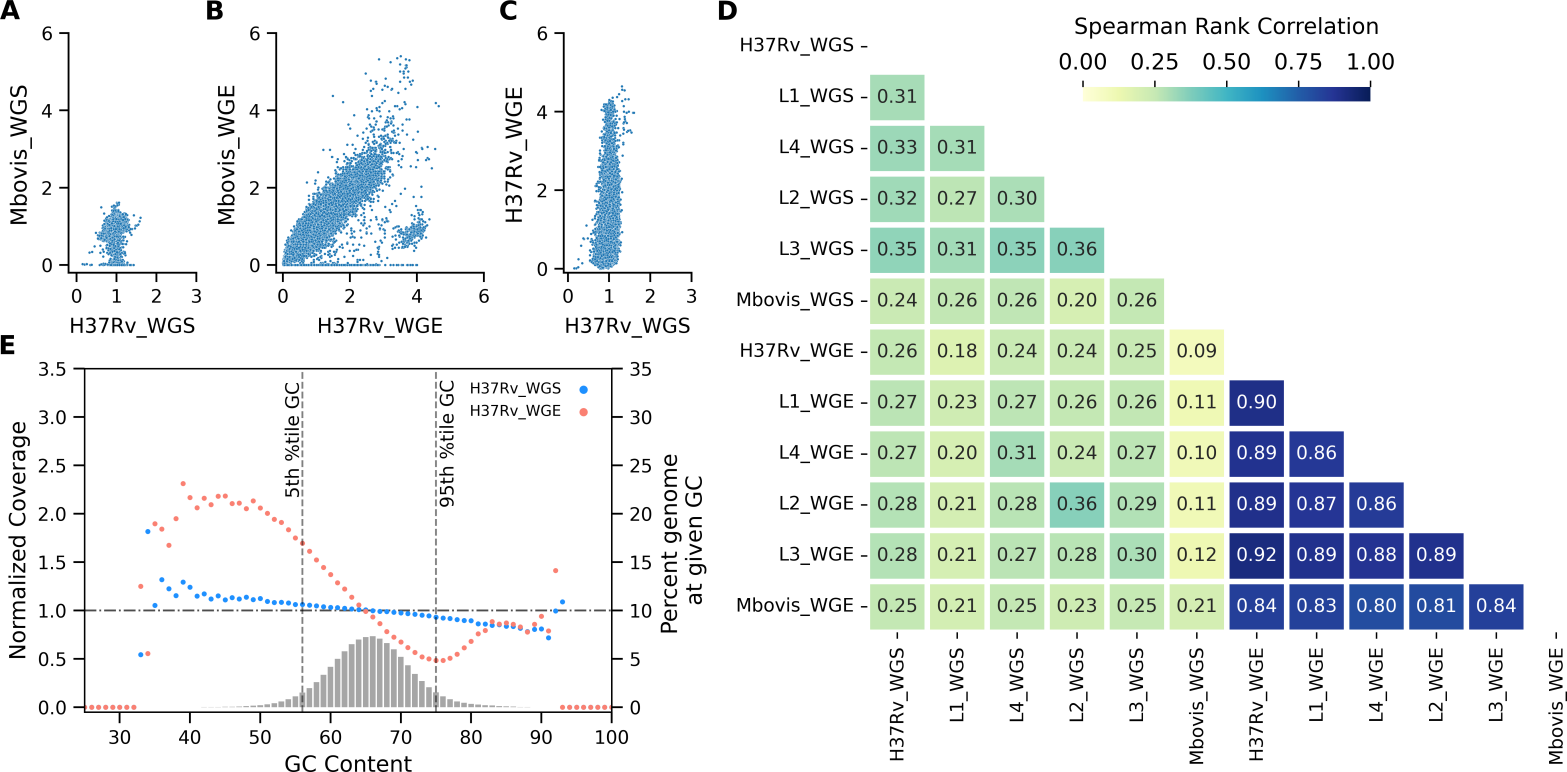
Correlation of normalized coverage at 100 genomic bins between WGS and WGE-CNERs data. Scatter plot of normalized coverage between *M. tuberculosis*-H37Rv and *M. bovis* generated by WGS (**A**) and WGE-CNERs (**B**). (**C**) Scatter plot of normalized coverage from WGS vs WGE-CNERs for *M. tuberculosis*-H37Rv 10,000-copy sample. (**D**) Heat map of Spearman rank correlations of pairwise comparisons of normalized coverage between WGS and WGE for five *M. tuberculosis* lineages and *M. bovis*. (**E**) Scatter plot of normalized coverage (primary y-axis) across G + C bins and histogram (secondary y-axis) of percentage of G + C bins plotted for WGS (cyan) and WGE-CNERs (orange) data for *M. tuberculosis*-H37Rv. The horizontal line shows normalized coverage at 1, and two vertical lines show the 5th and 95th percentile G + C bins of the genome.

We generated shotgun WGS data using the same mycobacterial DNA samples that produced 68× average genome coverage from 3 million raw read pairs. We normalized coverage for 100-bp genomic bins to account for differences in absolute coverage to compare WGS with WGE-CNERs. WGS resulted in uniform coverage across the 100 bp bins, resulting in normalized coverage closer to 1 (Fig. 3A; Fig. S9). The normalized coverage differed between lineages and species at specific bins due to the expected differences within the investigated genomic regions between different strains and lineages of MTBC (Fig. 3A; Fig. S9). WGE-CNERs data also reproduced the difference between lineages and species (Fig. 3B; Fig. S10). However, normalized coverage for different genomic loci varied up to five-fold within a sample (Fig. 3B and C; Fig. S11), indicating uneven coverage in some regions compared to more uniform coverage in WGS (Fig. 3C; Fig. S9). Pairwise comparisons of WGS coverage between MTBCs show a weak correlation with an average Spearman rho of 0.29 (upper triangle, Fig. 3D) but a strong correlation between WGE-CNERs experiments with an average Spearman rho of 0.86 (lower triangle, Fig. 3D). Genomic differences between MTBCs compounded with uneven coverage in WGE-CNERs resulted in a lower correlation between WGS and WGE-CNERs for different mycobacteria (center block, Fig. 3D). However, WGS and WGE-CNERs for the same mycobacteria were correlated with an average Spearman rho of 0.28 (center diagonal, highlighted, Fig. 3D), similar to the WGS correlations. We determined the normalized coverage across G + C bins using Picard *CollecGCBias* to check the effect of G + C content on coverage. For 90% of genomes with G + C content of 57%–75%, centered at 65% mean, the coverage differed ~6% from the mean coverage in WGS but differed ~55% from the mean coverage in the WGE-CNERs (Fig. 3E; Fig. S12). For the 10% of the genome with extreme G + C (<57% and >75%), the normalized coverage varied 0.5–2.5-fold in the CENRs-WGE (Fig. 3E; Fig. S12).

### Detection of MTBC lineages and durg resistance determinants from WGE-CNERs data

**FIG 4:**
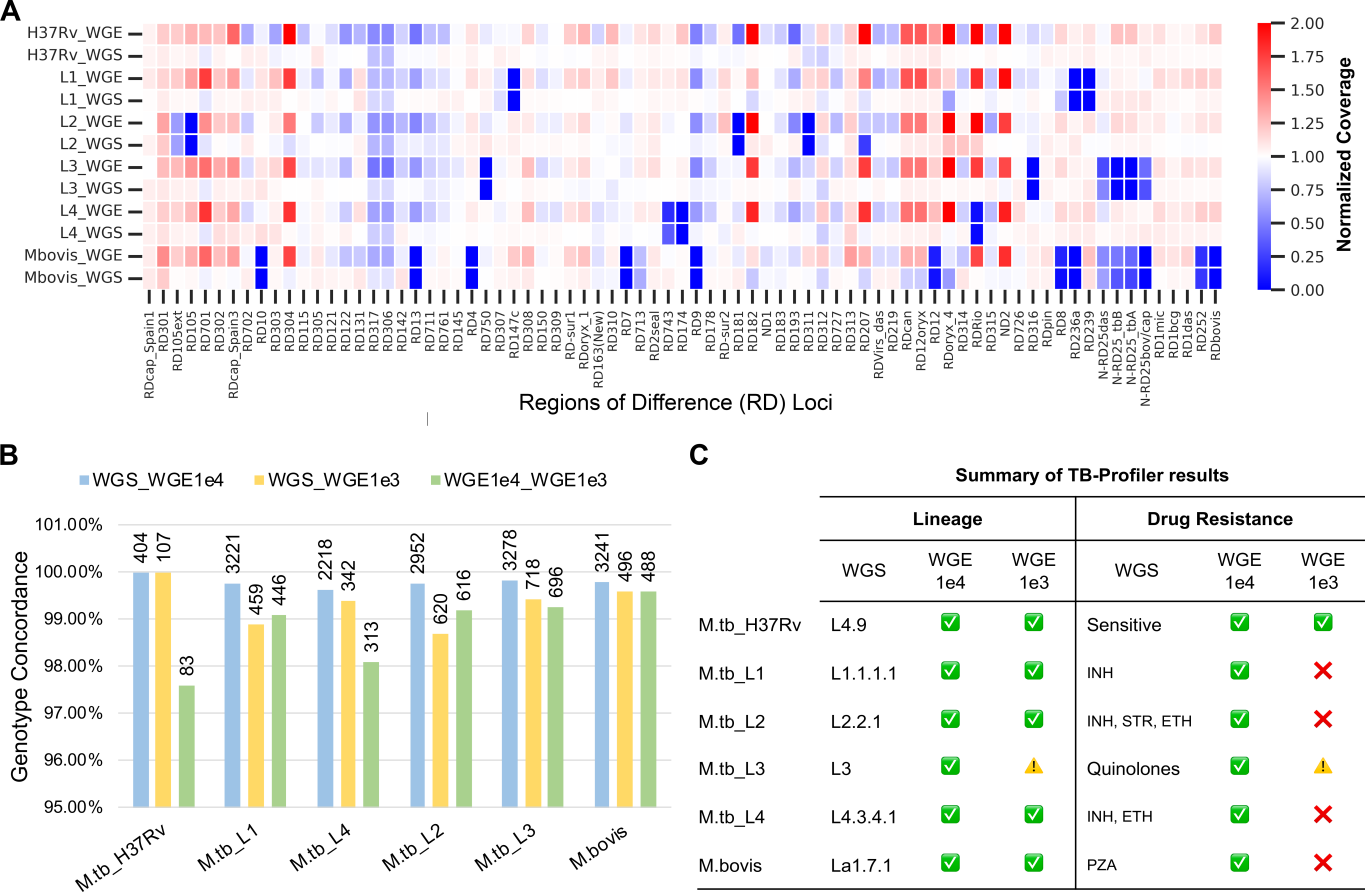
WGE-CNERs data can be used to genotype MTBC. (**A**) Heatmap of normalized coverage from WGS and WGE data for the five *M. tuberculosis* lineages and *M. bovis* at RD loci sorted by their genomic location. (**B**) SNP genotype concordance between WGS vs WGE-1e4 (cyan), WGS vs WGE-1e3 (yellow), and WGE-1e4 vs WGE-1e3 (green). Labels on top of the bar show the number of overlapping genotyped sites. (**C**) Summary of the TB-Profiler results. A green tick mark indicates agreement of TB-Profiler results between WGS and WGE for lineage and drug-resistance pattern. A red x-mark or caution indicates misclassification or no-classification of lineage and drug-resistance pattern in the WGE-1e3 data compared to WGS.

Regions of Difference (RD) loci are genome-wide small insertions and deletions specific to individual MTBC samples that are used for clinical strain typing (35). We plotted normalized coverage at RD loci as a heatmap to determine the coverage. WGS data identified RD deletions specific to each MTBC where coverage is zero (blue boxes, Fig. 4A). The 10,000-copy WGE-CNERs data also identified RD locus deletions where coverage is zero (blue boxes, Fig. 4A; Fig. S13A), but without G + C-based coverage normalization, the heatmap showed many RD loci with two-fold coverage (Fig. S13A). We normalized coverage based on G + C contents to eliminate most coverage unevenness in WGE-CNERs data. Yet, some RD loci appear to have two-fold coverage only in WGE-CNERs (red boxes, Fig. 4A). The 1,000-copy mixture WGE-CNERs data also identified RD locus deletions (Fig. S13B). Due to G + C coverage difference, WGE-CNERs data can only be used to discern deletions that are consistent with the WGS data but not for tandem duplications.

Sequencing data are used for strain typing and drug resistance profiling in genomic epidemiology. We used GATK HaplotypeCaller with the ploidy = 1 option to call variants, removed indels, and filtered for a minimum read depth of five reads in WGS and WGE data. After filtering, we genotyped 5,444 sites for the six *M. tuberculosis* lineages using WGS data, 5,416 sites using the 10,000-copy mixture, and 4,188 sites using the 1,000-copy mixture WGE-CNERs data (Table S2). We calculated genotype concordance as the percentage of genotypes (both reference and alternative alleles) that matched between WGS and WGE CNERs data over the total number of genotyped positions. Among the 2,552 overlapping positions between WGS and 10,000-copy WGE-CNERs data, 99.80% concur on average for five *M. tuberculosis* lineages and *M. bovis* (Fig. 4B, cyan bars and Table S2), 99.35% of 457 overlapping sites concur between WGS and 1,000-copy WGE-CNERs data (Fig. 4B, yellow bars and Table S2), and 98.81% of 440 overlapping sites concur between 1,000-copy and 10,000-copy WGE-CNERs data (Fig. 4B, green bars and Table S2).

Among 34 drug-resistance conferring genes (Table S3), we found on average 36 variants (14–45) per sample that concurred 100% between the WGS and 10,000-copy mixture WGE-CNERs data. To characterize variants in drug-resistance conferring genes and identify the drug-resistance pattern, we used the TB-Profiler (36) web tool. WGS and 10,000-copy WGE-CNERs data correctly identified expected lineages, both of which also matched for all six samples using TB-Profiler (Fig. 4C; Table S4). Lineages for five of six samples were also correctly identified using the 1,000-copy WGE-CNERs data. Lineage was not determined for the L3 sample using 1,000-copy WGE-CNERs data, which might be due to low coverage. TB-Profiler also identified the drug-resistance patterns using variants identified in WGS and 10,000-copy WGE-CNERs. Both data identified that *M. tuberculosis*-H37Rv is sensitive to all drugs; L1 is resistant to isoniazid due to the Ser315Thr missense mutation in KatG; L2 is resistant to three first-line drugs (isoniazid, streptomycin, and ethionamide); L3 is resistant to all quinolones due to the Ser91Pro missense mutation in Gyrase A; L4 is resistant to isoniazid and ethionamide; and *M. bovis* is resistant to pyrazinamide due to the His57Asp missense mutation in PncA (Fig. 4C; Table S4). Due to the coverage cutoff of 10 reads in the TB-Profiler pipeline to assign variants, four samples were misclassified as sensitive to all drugs, and the L3 sample was misclassified as resistant to ethionamide using the 1,000-copy WGE-CNERs data (Fig. 4C; Table S4).

## DISCUSSION

We demonstrated that the CNERs method can generate microgram quantities of WGE baits that can be used to enrich MTBC DNA for genomic analyses. We spiked a wide range of MTBC genome copies with a constant amount of human DNA to mimic clinical sputum samples. Our results illustrated that WGE-CNERs can enrich *M. tuberculosis* DNA as low as 0.01% in the initial sample and as low as 100 absolute copies of MTBC genomes from a vast majority of human DNA backgrounds. We showed that the breadth and depth of genome coverage using WGE-CNERs depended on the copy number in the initial sample and after-enrichment sequencing depth, as previously observed for direct sequencing of clinical samples (10, 20 – 22, 37 – 39). Furthermore, we showed that short-duration (1–4 h) hybridization using WGE-CNERs can detect 1,000 or more bacilli, and that overnight (16–20 h) hybridization can detect as few as 100 tuberculosis bacilli. *M. tuberculosis* detection threshold for the sputum acid fast smear test is 5,000–10,000 bacilli/mL (40), and for the Xpert MTB method, it is ~100 bacilli/mL (40, 41). The detection threshold may vary based on the initial volume of sputum sample processed by these methods, which must be noted when comparing the detection threshold of WGE-CNERs enrichment with these methods.

Previous studies find higher concordance in drug-resistance genotypes between direct sequencing and WGS after culturing isolates (22, 37, 38). However, studies also identified higher genetic diversity and hetero-drug resistance from direct sequencing of clinical samples that are lost after culturing (10, 20). We demonstrated the WGE-CNERs method using contrived mixtures of mycobacterial DNA isolated from pure culture mixed with human DNA. Though the mixtures mimicked clinical specimens with human and pathogen DNA, they were lacking other microbiome DNA present in actual clinical samples. Future work is needed to evaluate how microbiome DNA present in various clinical samples may affect WGE-CNERs enrichment efficiency and specificity. In addition, further studies are needed to evaluate the concordance between predicted drug-resistance patterns identified from direct sequencing of clinical samples using WGE-CNERs enrichment and sequencing from pure culture isolates. We expect that the WGE-CNERs method may have utility in molecular drug susceptibility testing and would reduce time-to-results, which is currently a challenge using time-intensive culture-based methods.

Clinical samples with commensal microbiomes and complex bacterial mixtures may pose challenges in specific pathogen DNA enrichment due to the presence of non-target genomes with highly similar gene sequences, like rRNA and other conserved bacterial genes. Hybridization capture methods tolerate some sequence mismatch between probe and target (42). To experimentally prevent inadvertent co-enrichment of non-target bacteria, rRNA depletion methods like the addition of rRNA blocking oligos could be used (43). Alternatively, when analyzing enriched reads using reference-based mapping tools, conserved genes/regions and repeat regions can be masked to avoid mapping reads from non-target bacteria. This strategy has been used for designing WGE RNA-baits (10, 22). Furthermore, enrichment data can be analyzed using metagenomic tools like MEGAN (34) or Kraken (44) that search databases like NCBI with a wide collection of sequences rather than a specific reference to verify percent reads identified as target pathogens vs non-target bacteria, as we demonstrated in Fig. S7.

We also demonstrated that CNERs made using *M. tuberculosis* DNA as a template specifically enriches MTBC genomes and poorly enriches NTM genomes similar to the RNA baits previously used (38). NTMs cause pulmonary disease and may coinfect MTBC in tuberculosis-endemic regions. Differentiation between MTBC and NTMs is necessary for clinical decisions as both manifest as acid-fast bacilli in clinical smear testing (45, 46). It would be interesting to generate NTM-specific WGE-CNERs for use in combination with *M. tuberculosis* CNERs for a unified WGE panel to capture a variety of primary pathogenic mycobacteria.

It would be important to create a WGE panel that would allow for the capture of the pangenomes of all MTBC. Previous studies designed RNA baits using ~3,650 MTBC genomes (21) or an ancestral *M. tuberculosis* sequence that is equidistance to all lineages (37) for pangenome enrichment. A large number of custom-made RNA baits would be needed to target multiple lineages for pangenome coverage. Customizing and making RNA baits is expensive, though it allows for the inclusion of important regions like pangenome regions and antimicrobial resistance (AMR) genes and the exclusion of redundant genes like rRNA. The prohibitive cost of custom-made RNA baits limits the WGE approach to detecting novel and difficult-to-grow pathogens at large scales during routine surveillance and pandemics. Bioinformatic design of custom RNA baits also requires high-quality reference genomes (22, 30, 31). These requirements limit the ability to sequence novel pathogens and new variants using custom-made baits. The CNERs method does not require prior genome sequence information but requires purified DNA from the pathogen of interest or a closely related species. CNERs, like all hybridization capture methods, can hybridize and enrich for DNA sequences that are similar to the CNER probes. This facilitates CNERs ability to capture the core genome sequences common among the lineages and subspecies without the need for customized baits and enable high-resolution genotyping in the core genomic regions for phylogenic studies. Furthermore, the simplicity of CNER probe production allows the use of a mixture of gDNA templates from diverse strains and lineages that would be a cost-effective alternative to custom-made baits for pangenome enrichment. Future studies must address how mixing template gDNA from many lineages affects the CNER generation, WGE efficiency, and coverage differences in the core vs unique genomic regions of individual strains and lineages. Future studies must also address the feasibility of creating a diverse enough WGE panel to capture the pangenome of species that possess significantly more accessory genes (like *Escherichia coli*) than what is seen in *M. tuberculosis*.

Genome size, GC content, and large indel/copy number variations between strains might be limiting factors when adopting the CNERs method for other pathogens. We observed a five-fold coverage difference between different GC regions in *M. tuberculosis*. Repeat elements and conserved genomic regions among pathogens of the same family or phylum might also be limiting factors when adopting CNERs for pathogens with large genomes. However, we have demonstrated the utility of the WGE-CNERs approach for such pathogens by making CNERs against *Toxoplasma gondii* (under review in *Food Microbiology*).

WGE is more cost-effective than the shotgun WGS approach to sequencing pathogen genomes directly from clinical samples. However, expensive custom-made baits prohibit this approach for the large-scale sequencing needed for genomic epidemiology. Previous WGE methods also required a longer turnaround time (TAT) due to overnight hybridization, which is critical during epidemics (25). Hybridization time can be shortened for CNERs probes to reduce TAT. Furthermore, the CNERs method produces microgram quantities of probes that would make it cost-effective. At the time of publication, we estimated about $100 in reagent costs to make CNERs per capture reaction (Table S5); however, we did not estimate the labor cost as it would widely vary. Assuming an equal cost for labor and reagents, CNERs will cost about $200 per capture reaction, which is ~40% less than the SureSelect RNA baits used in previous studies to enrich *M. tuberculosis* (22, 47). These advantages make CNERs an alternative to custom-made RNA baits for WGE sequencing needed for genomic epidemiology applications.

The CNERs method described here can be extended to culture-independent diagnostic tests (CIDTs) for other difficult-to-grow pathogens. Current (q)PCR-based CIDT methods offer rapid detection of pathogens but provide only limited detection of AMR determinants. CIDTs also fail to provide genotype data for clinical isolates, which may impede outbreak surveillance. Furthermore, laboratories that implement CIDTs for foodborne pathogens tend to skip bacterial isolation and culturing that are required for surveillance networks using WGS pipelines for strain typing (48, 49). We propose WGE-CNERs as an alternative method that can provide the benefits of both culture-based WGS and CIDT methods. We envision the WGE-CNERs approach being adopted not only for rapid detection as a CIDT but also for routine genomic surveillance to characterize AMR patterns, to detect emerging clinical strains and lineages, and to predict outbreaks of a wide range of microbial pathogens.

## MATERIALS AND METHODS

### DNA samples and library preparation

The California Department of Public Health’s Center for Laboratory Sciences Microbial Diseases Laboratory kindly provided mycobacterial gDNA, including gDNA prepared from the American Tissue Culture Collection *M. tuberculosis-*H37Rv. We prepared NGS libraries using the NEB Ultra II FS kit, following the manufacturer’s instructions.

For the proof-of-concept experiment, we intentionally spiked *M. tuberculosis*-H37Rv libraries with unique dual indices at 0.01%, 0.1%, 1%, and 10% expected representation with human libraries (prepared with NA12878 gDNA). We sequenced the contrived mixture of *M. tuberculosis* and human libraries to confirm the proportion of *M. tuberculosis* libraries before capture.

To determine sensitivity, we quantified gDNA using the Qubit HS kit (Thermo Fisher) and spiked 10 fg–95.2 ng (eight times 1:10 serial dilution) of *M. tuberculosis-*H37Rv gDNA corresponding to 2 × 10^0^–2 × 10^7^ *M. tuberculosis* genome copies with 54 ng of human gDNA (NA12878) and prepared NGS libraries using the NEB Ultra II FS kit with the following modifications. After fragmentation and adapter ligation, we split the adapter-ligated DNA into two aliquots (corresponding to 1 × 10^0^–1 × 10^7^ *M. tuberculosis* genome copies per library) and amplified them for 8 cycles with the NEB Q5 master mix with two sets of unique dual indices. We sequenced all 16 libraries before the capture experiments to determine the *M. tuberculosis* proportion in each library.

For the specificity test, we spiked 140 fg–142.8 fg (four times 1:10 serial dilution corresponding to 3 × 10^1^–3 × 10^4^ *M. tuberculosis* genome copies) of four *M. tuberculosis* lineages (*M. tb* Indo-Oceanic [L1] , East-Asian [L2], East-African-Indian [L3], and Euro-American [L4]), *M. bovis*, and three NTMs (*M. abscessus*, *M. fortuitum*, and *M. porcinum*) with 54 ng of human gDNA (NA12878) and prepared NGS libraries using the NEB Ultra II FS kit with the following modifications. After fragmentation and adapter ligation, we split the adapter-ligated DNA into three aliquots (corresponding to 1 × 10^1^–1 × 10^4^ *M. tuberculosis* genome copies per library) and amplified them for 12 cycles with the NEB Q5 master mix with three sets of unique dual indices. We sequenced all 96 libraries before capture experiments to determine the proportion of mycobacteria in each library.

### *M. tuberculosis* WGE-CNERs generation

We generated WGE-CNERs to enrich mycobacteria as described in Fig. S1. We sheared ~286 ng of *M. tuberculosis*-H37Rv reference gDNA using Covaris microTUBE-15 for 250 s with peak power at 50, 30% duty factor, and 50-cycle bursts at 23°C. We denatured 100 ng of sheared gDNA at 95°C for 3 min and snap-cooled it on an ice block. Separately, 100 pmol of bridge oligo (5′-GCGCGATCAAGCTTTTTTTTTTTTTTTTTTTTT-3′) was annealed with splint oligos (5′-NNNNNNNNAAAAAAAAAAA-3′ and 5′-GCTTGATCGCGCNNNNNNNN-3′) by denaturing at 95°C for 3 min and cooling to 12°C with 0.1°C/s ramp speed. Sheared denatured gDNA was mixed with 35 pmol of annealed bridge/splint oligos and ligated in 1× T4 DNA ligase buffer at 37°C for 1 h, followed by 25°C for 3 h, and denatured at 95°C for 3 min. We amplified the circularized genomic fragments as described in Sundararaman et al. (32) and digested the RCA products using 50 U of HindIII enzyme.

### Enrichments and sequencing

For the proof-of-concept experiment, we hybridized 100–300 ng of four contrived mixtures of *M. tuberculosis* and human libraries with 50 ng of *M. tuberculosis* WGE-CNERs at 65°C for 19.5 h. For the sensitivity test, we hybridized 100 ng of eight individual libraries of various *M. tuberculosis* copy numbers with 25 ng of WGE-CNERs or pooled 25 ng from each of the eight libraries and hybridized the pool with 50 ng of WGE-CNERs.

For the specificity test, we pooled two sets of 11 ng each of the *M. tuberculosis*-H37Rv, four *M. tuberculosis* lineages, *M. bovis*, and three NTM libraries from the same copy number mixture of 1 × 10^1^–1 × 10^4^ copies. For the hybridization temperature experiment, we captured the pools with 25 ng of WGE-CNERs at 55°C and 60°C for 19.5 h. For the hybridization time experiment, we captured the pools with 25 ng of WGE-CNERs at 65°C for 1 and 4 h. For captures at 65°C, we pooled 12.5 ng each of the four lineages, *M. bovis*, and three NTMs from the same copy number libraries.

For all capture experiments, we enriched the captured library with streptavidin beads as described in Sundararaman et al. (32). We amplified post-capture libraries with a 2× Kapa HiFi PCR mix for 17 cycles and purified the libraries using 1.2× SPRI beads. Post-capture libraries were pooled in an equimolar ratio and sequenced in the Illumina NextSeq with a PE 2× 75 kit. For all experiments, we sequenced ~50k–100k raw read pairs. We sequenced ~3 million raw read pairs for each of the libraries captured at 65°C for 19.5 h.

### Data analysis

We used Cutadapt v3.5 (50) to remove adapter sequences and mapped the trimmed reads to *M. tuberculosis*-H37Rv reference genome (NC_000962.3) using bwa mem v0.7.17-r1188 (51). We used samtools v1.6 rmdup (52) to remove duplicate reads. We used samtools and bedtools v2.29.1 (53) to determine the percent mapped reads and genome coverage. We used custom Python scripts (https://github.com/bsun210/WGE_CNERs_Mtb_pathogen_genomics) to plot the metrics. We performed a non-parametric Mann-Whitney *U* rank test using a python package-statannot (https://github.com/webermarcolivier/statannot) for comparing coverage metrics between different hybridization temperatures and times.

We blast searched 50,000 raw reads using locally installed BLASTn v2.10 (54) against the NCBI nucleotide (nt) database v5 (33) and analyzed the results using Metagenomic Analyzer (MEGAN) software v6.22.2 (34) to independently check the identity of the sequencing reads.

For variant detections, we used HaplotypeCaller from the GATK v4.2.6 package (55) with -ERC GVCF and -ploidy 1 options to individually call variants on each sample from 1,000- to 10,000-copy numbers captured at 65°C for 19.5 h. We used CombineGVCFs and GenotypeGVCFs with default options to combine Variant Call Format files (VCFs) from five *M. tuberculosis* lineages and *M. bovis* of the same copy number. We used vcftools v0.1.17 with --max-alleles– 2 --remove-indels options to remove indels and filter for biallelic variants. We also used --min-meanDP 5 to filter for read depth. We used bcftools stats to determine genotype concordance between variants from 1,000- to 10,000-copy number samples.

We used the TB-Profiler (36) web tool v4.4.1 to identify the lineages and drug-resistance-conferring mutations. Run IDs for each sample are provided in Table S4.

## Data Availability

All raw sequencing data generated for this project are available in the NCBI SRA database under BioProject accession number PRJNA946035. The bioinformatic data processing pipeline and custom Python scripts used for making figures are available on the GitHub page https://github.com/bsun210/WGE_CNERs_Mtb_pathogen_genomics.

## SUPPLEMENTAL MATERIAL

Supplemental Figures mbio.01889-23-s0001.pdf Figures S1 to S13.

Supplemental Tables mbio.01889-23-s0002.xlsx Tables S1 to S5.

ASM does not own the copyrights to Supplemental Material that may be linked to, or accessed through, an article. The authors have granted ASM a non-exclusive, world-wide license to publish the Supplemental Material files. Please contact the corresponding author directly for reuse.
