# Supplementary material for: A hybridization target enrichment approach for pathogen genomics
Source: mBio. 2023 Oct 13;14(5):e01889-23. doi: 10.1128/mbio.01889-23 (PMC10653935; doi:10.1128/mbio.01889-23)
Supplement: Supplemental Figures — Figures S1 to S13. [file mbio.01889-23-s0001.pdf]

A hybridization capture approach for pathogen genomics

Balaji Sundararaman,[a]# Matthew D. Sylvester[b], Varvara K. Kozyreva[b], Zenda L. Berrada[b], Russell B Corbett-Detig[a,c] and Richard E. Green[a,c]#

Supplementary Figures

Figure S1: *M. tuberculosis* Whole Genome Enrichment CNERs generation.

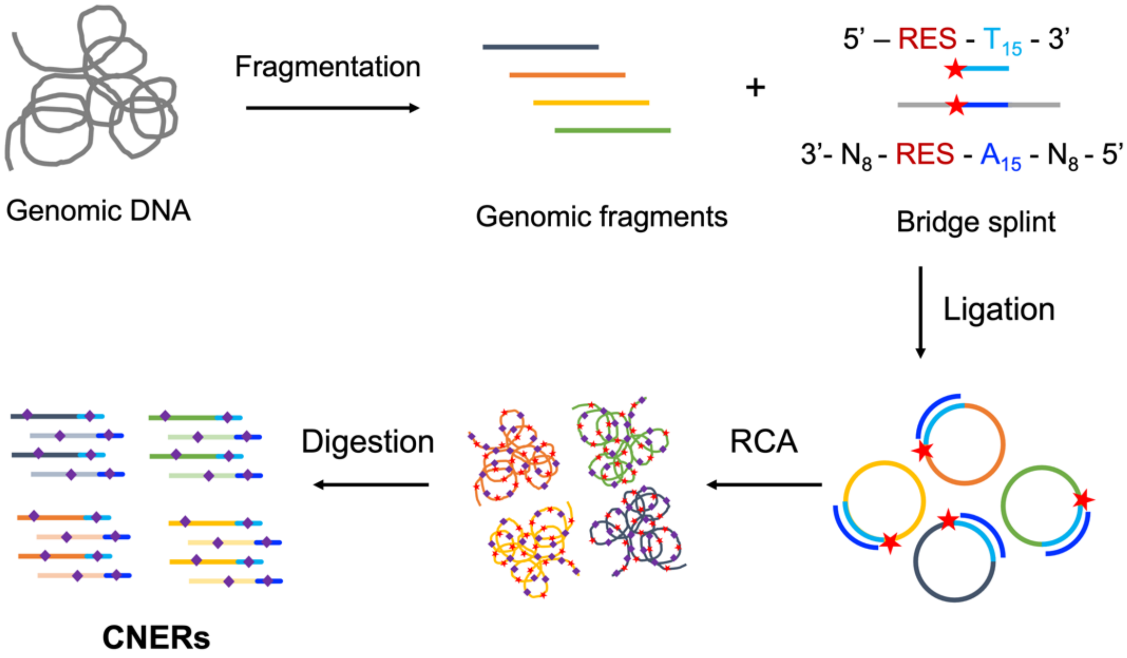

Figure S1: Whole Genome Enrichment *C*ircular *N*ucleic acid *E*nrichment *R*eagent synthesis method. Genomic DNA of target pathogen is sheared to generate gDNA fragments. Genomic fragments are ligated to a bridge adapter with an upper oligo containing restriction enzyme recognition site (RES) and oligo-dT and a bottom oligo complimentary to the upper oligo with degenerate nucleotides (N) in both ends. These degenerate nucleotides complement the ends of gDNA fragments to facilitate head-tail circularization which also ligates the upper oligo. Circularized templates are isothermally amplified using oligo-dA (blue) and oligo-dT (cyan) oligos by rolling circle amplification (RCA). RCA products are then digested with restriction enzymes to generate CNERs. CNERs generate both strands (dark and light shades of colors) of the templates. Biotinylated nucleotides (purple diamonds) are incorporated during amplification. Hence, CNERs can be used for hybridization capture of target DNA molecules on streptavidin coated beads.

Figure S2: *M. tuberculosis* Whole Genome Enrichment CNERs generation.

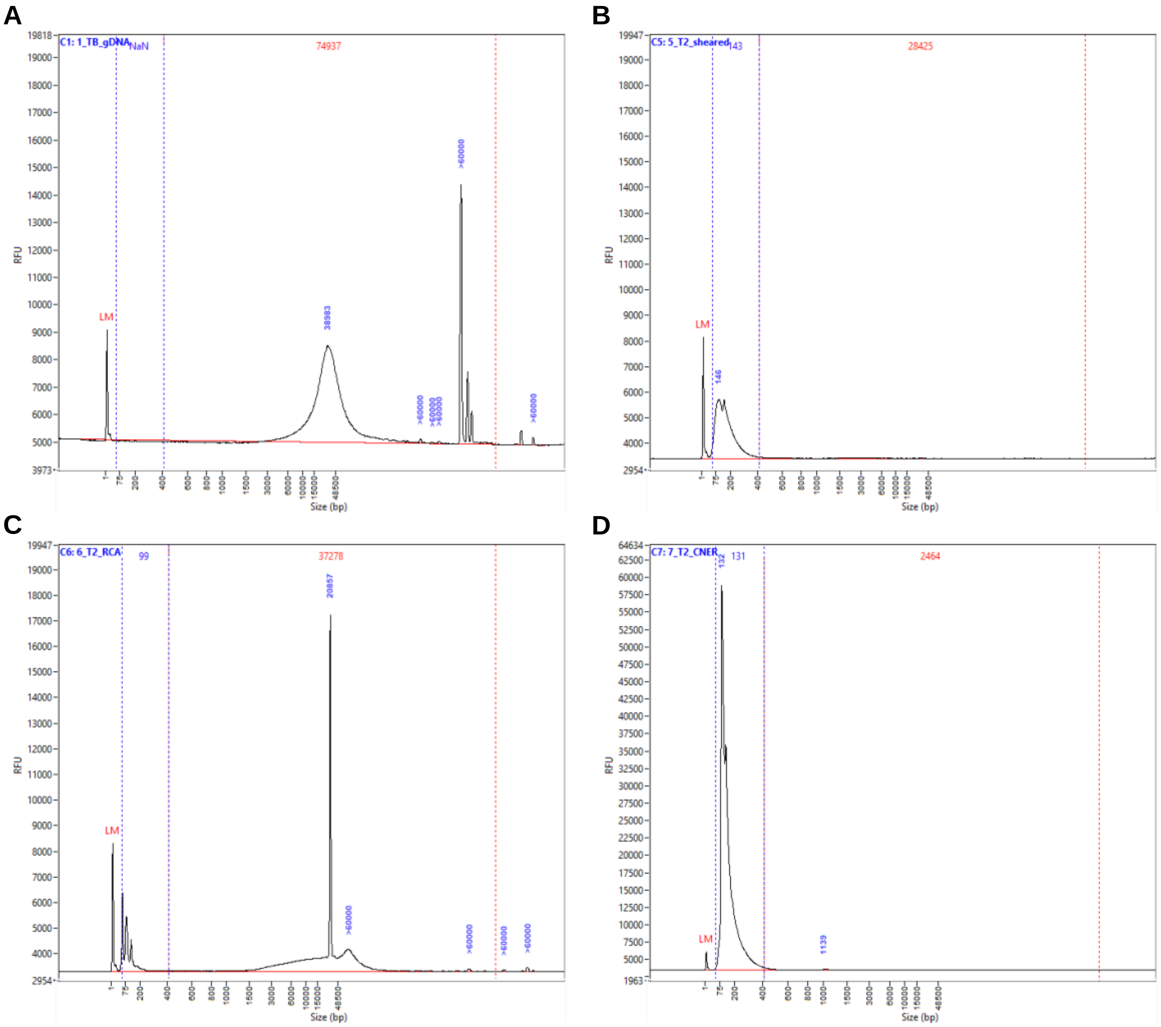

Figure S2: *M. tuberculosis* Whole Genome Enrichment CNERs generation. (A) Capillary electrophoretogram generated using Fragment Analyzer genomic DNA kit show the high molecular weight *M. tb* H37Rv genomic DNA with ~75kb mean size. **(B)** Covaris shearing produced genomic DNA fragments with 143bp mean size. **(C)** RCA amplification of circularized gDNA fragments produced high molecular weight DNA with >60kb size. **(D)** RCA products digested with HindIII restriction enzyme generated >99% monomeric CNERs with 131bp mean size.

Figure S3: Pairwise comparisons of normalized coverage for different copy mixtures

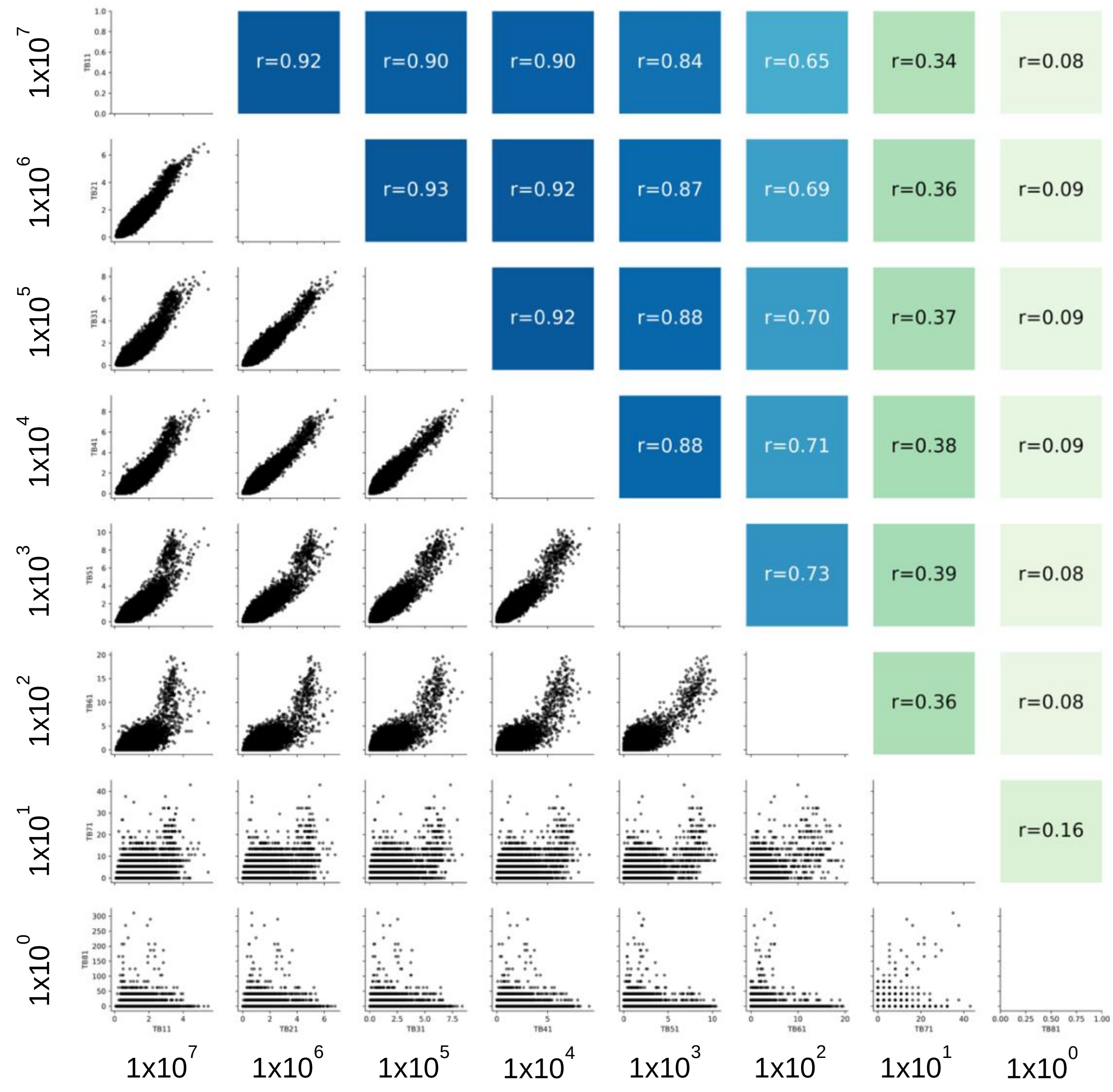

Figure S3: Pairwise comparisons of normalized coverage of 100 bp bins across the genome for the 8 *M. tuberculosis* copy mixtures enriched with WGE-CNERs. Normalized coverages are plotted as scatter plot between pairs of different copy mixtures and the corresponding Pearson's r value is shown in the upper triangle.

Figure S4: M. tb CNERs does not enrich non-tuberculosis Mycobacteria.

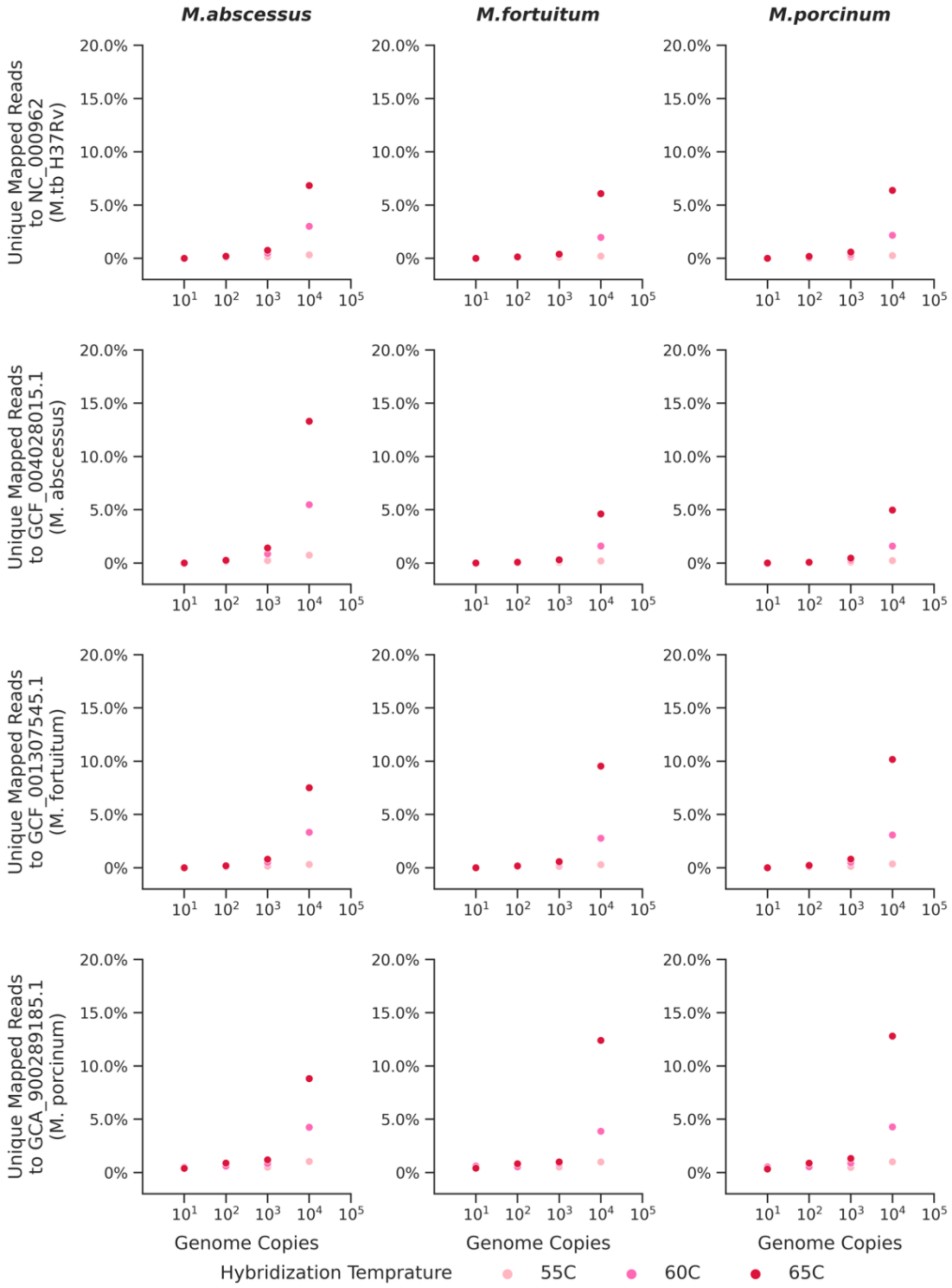

Figure S4: *M. tb* CNERs does not enrich non-tuberculosis Mycobacteria. Unique mapped reads to *M. tb* reference (NC_000962), *M. abscessus* reference (GCF_004028015.1), *M. fortuitum* reference (GCF_001307545.1) and *M. porcinum* reference (GCA_900289185.1) for the three NTM samples (columns) with 10 – 10,000 copy mixtures captured with *M. tb* WGE-CNERs at three different hybridization temperatures show that NTMs are poorly enriched by *M. tb* CNERs.

Figure S5: Percent unique mapped reads for individual MTBC captures.

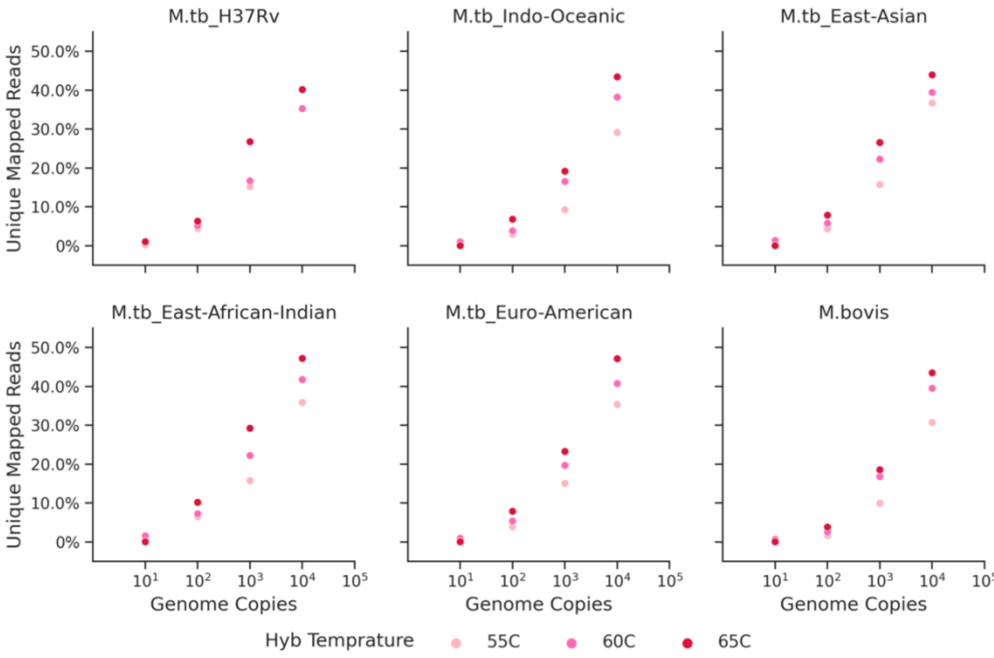

Figure S5: Percent unique mapped reads for individual MTBC captures. Unique mapped reads to *M. tb* reference (NC_000962) for the MTBC samples with 10 – 10,000 copy mixtures captured with *M. tb* WGE-CNERs show that increasing copy numbers increase the enrichment efficiency.

Figure S6: Fold enrichment for individual MTBC captures.

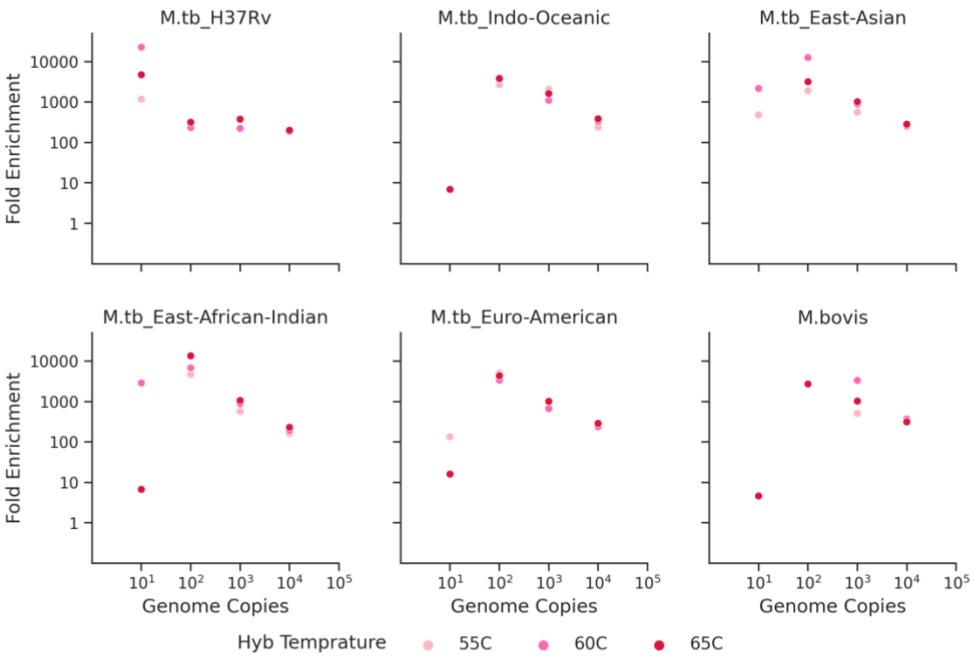

Figure S6: Fold enrichment for individual MTBC captures. Fold enrichment based on the unique mapped reads to *M. tb* reference for the MTBC samples with 10 – 10,000 copy mixtures captured with *M. tb* WGE-CNERs show that increasing copy numbers decrease the fold-enrichment.

Figure S7: MEGAN analyses of MTBC WGE-CNERs data.

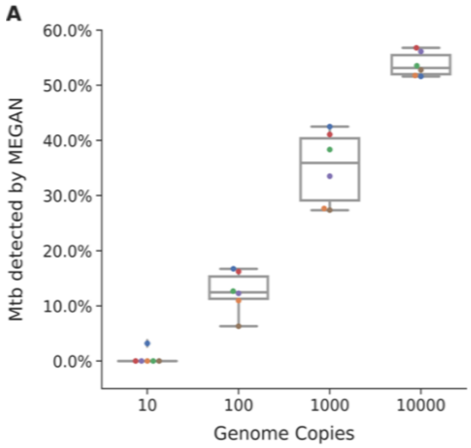

Figure S7: MEGAN analyses of MTBC WGE-CNERs data. Boxplots percentage of reads assigned as *M. tuberculosis* by MEGAN analysis of *blastn* search results for the 50,000 subsampled raw reads from the WGE-CNERs data generated by enrichments at 65°C for 19.5hr for the six MTBCs at four genome copy numbers.

Figure S8: Genome coverage from WGE-CNERs data for four *M. tuberculosis* lineages.

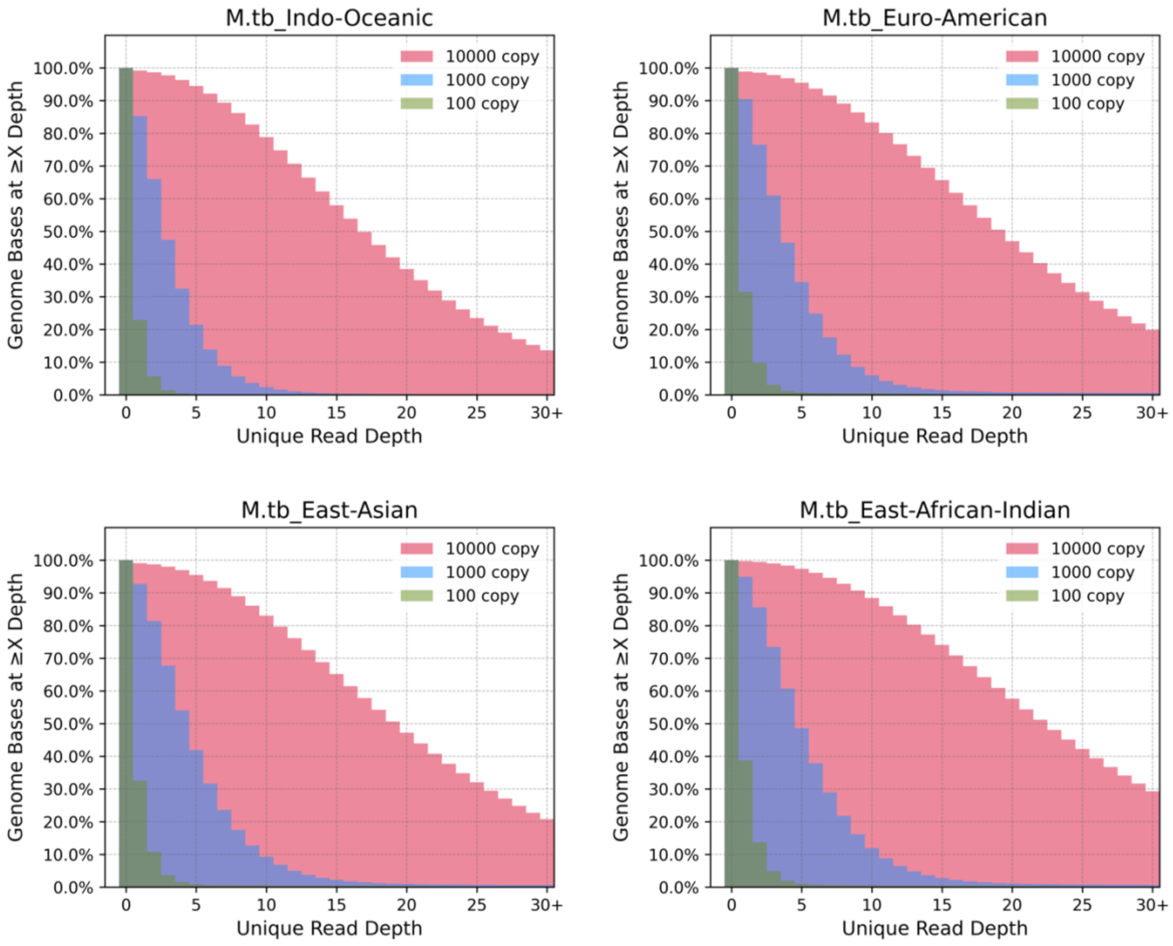

Figure S8: Genome coverage from WGE-CNERs data for four *M. tuberculosis* lineages. Overlapping histogram of percent of genome with X or more unique read depth from three million reads of WGE-CNERs data for 10,000-copy (pink), 1,000-copy (cyan) and 100-copy (green) mixtures of four *M. tuberculosis* lineages.

Figure S9: Pairwise scatterplots of normalized coverage generated by WGS for MTBCs

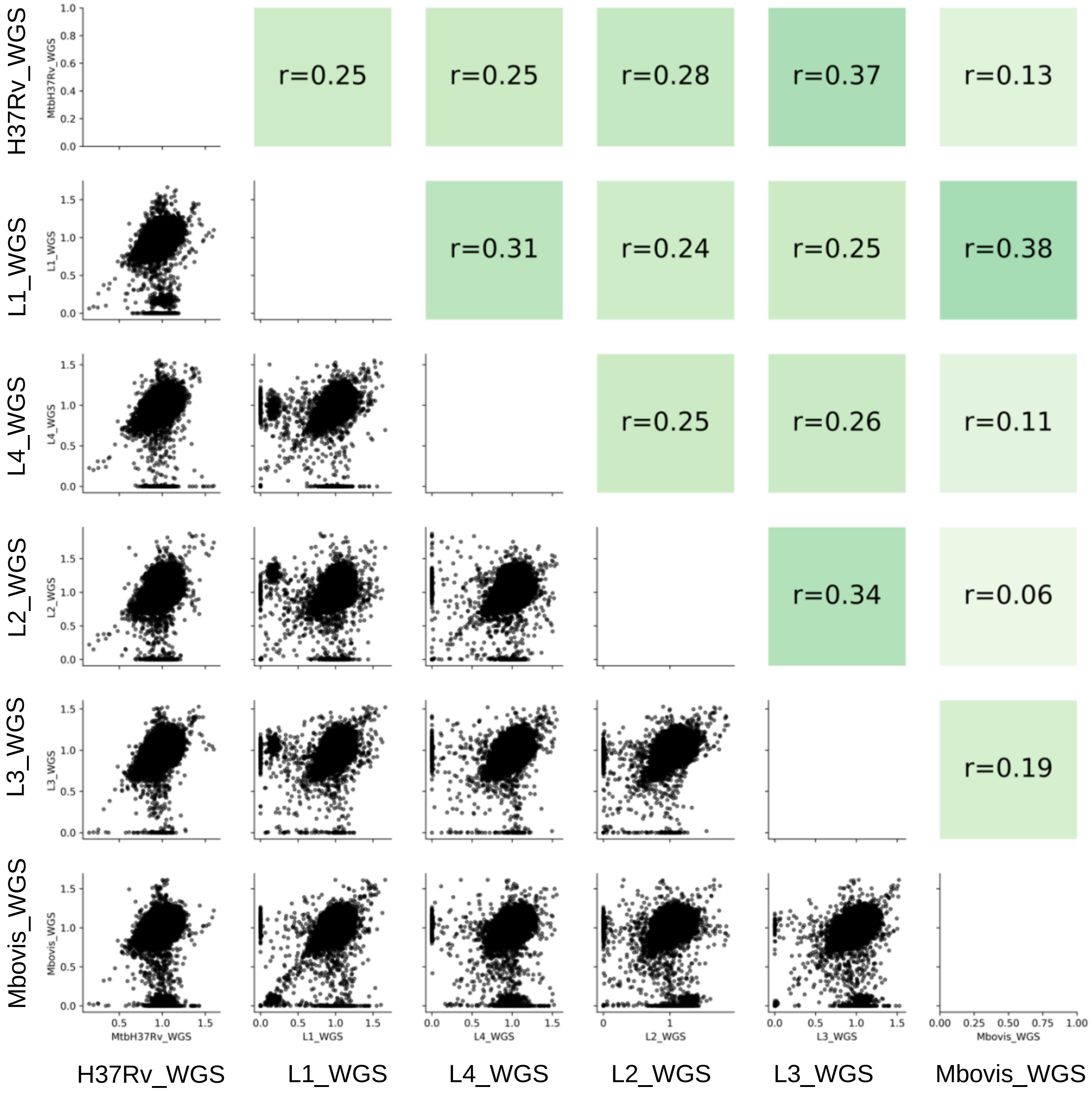

Figure S9: Pairwise scatterplots of normalized coverage generated by WGS for MTBCs. Scatter plots and heat map of Pearson's r correlation of pairwise comparisons of normalized coverage between six MTBCs for WGS data. Axes scale are <2 for normalized coverage.

Figure S10: Pairwise scatterplots of normalized coverage generated by WGE-CNERs for MTBCs

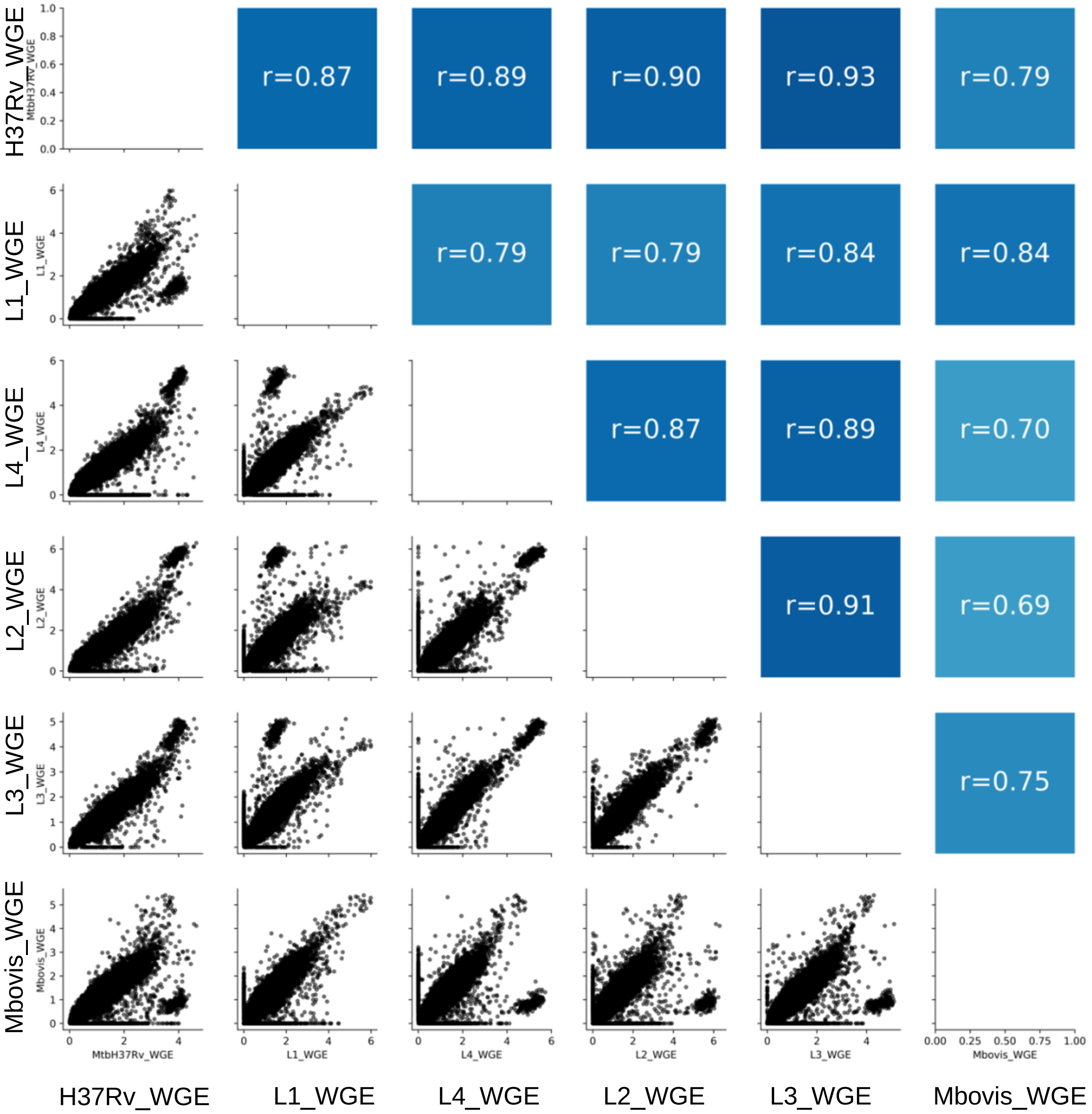

Figure S10: Pairwise scatterplots of normalized coverage generated by WGE-CNERs for MTBCs. Scatter plots and heat map of Pearson's r correlation of pairwise comparisons of normalized coverage between six MTBCs for WGE-CNERs data. Axes scale are <6 for normalized coverage.

Figure S11: Pairwise comparison of normalized coverage between WGS and WGE-CNERs

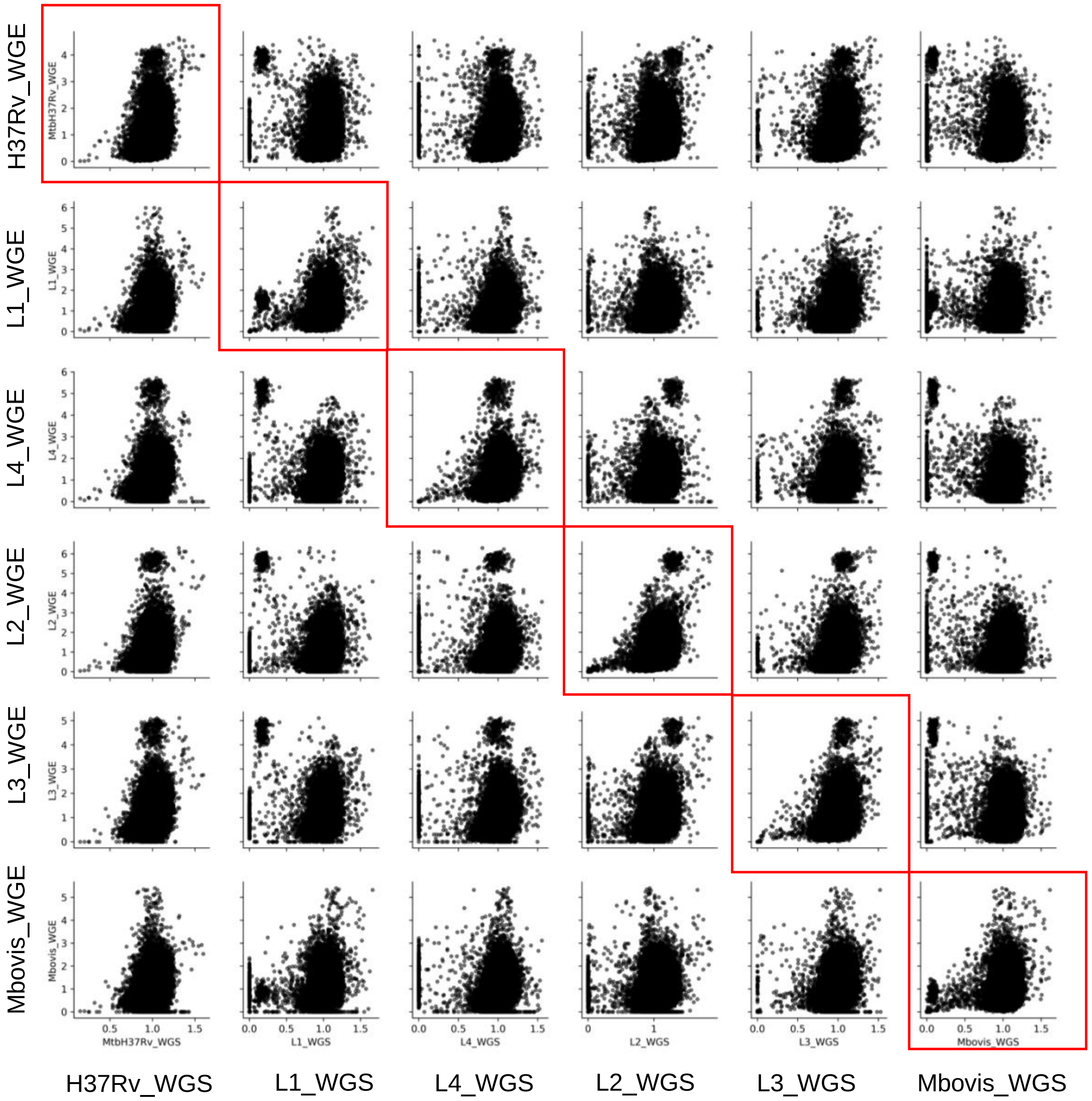

Figure S11: Pairwise comparison of normalized coverage between WGS and WGE-CNERs. Scatter plots of pairwise comparisons of normalized coverage between six MTBCs for WGS and WGE-CNERs data. Y-axis scales are <6 and X-axis scales are <2 for normalized coverage. Same sample comparison between WGS vs WGE-CNERs in the diagonal are highlighted in red. The heat map of Spearman rank correlations is shown in main Figure 3D.

Figure S12: Scatter plot of normalized coverage across G+C bins for WGS and WGE-CNERs

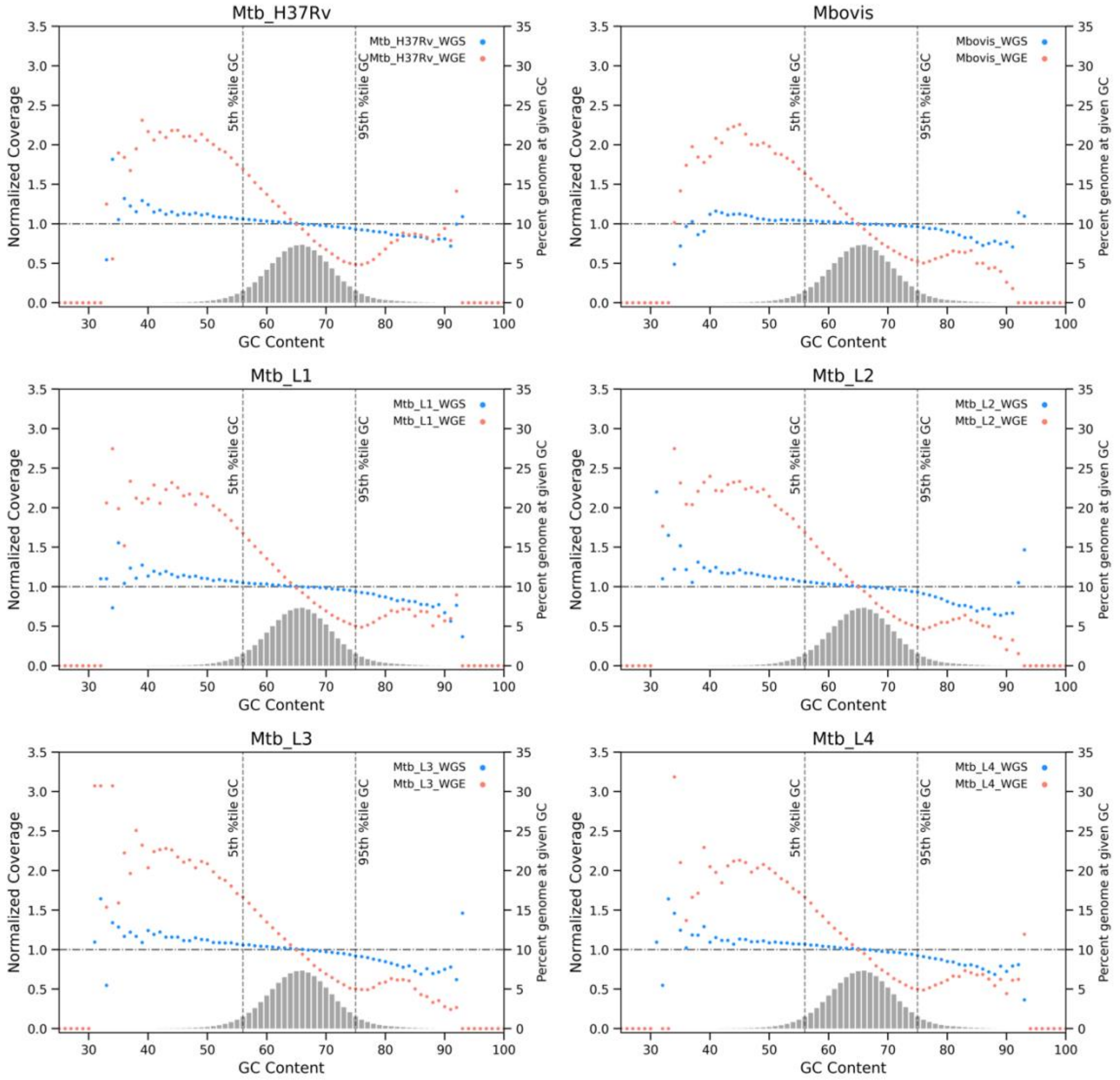

Figure S12: Scatter plot of normalized coverage across G+C bins for WGS and WGE-CNERs. Scatter plot of normalized coverage (primary Y-axis) across G+C bins and histogram (secondary Y-axis) of percentage of G+C bins plotted for WGS (cyan) and WGE-CNERs (orange) data for six MTBCs. Horizontal line show normalized coverage at 1 and two vertical lines show the 5th and 95th percentile G+C bins of the genome.

Figure S13: Heatmap of raw normalized coverage at RD loci from 10,000- and 1000-copy data

A

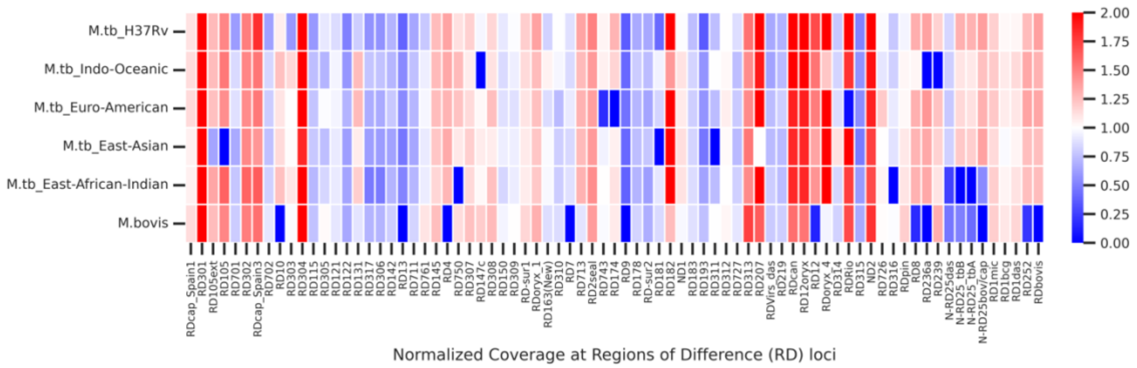

B

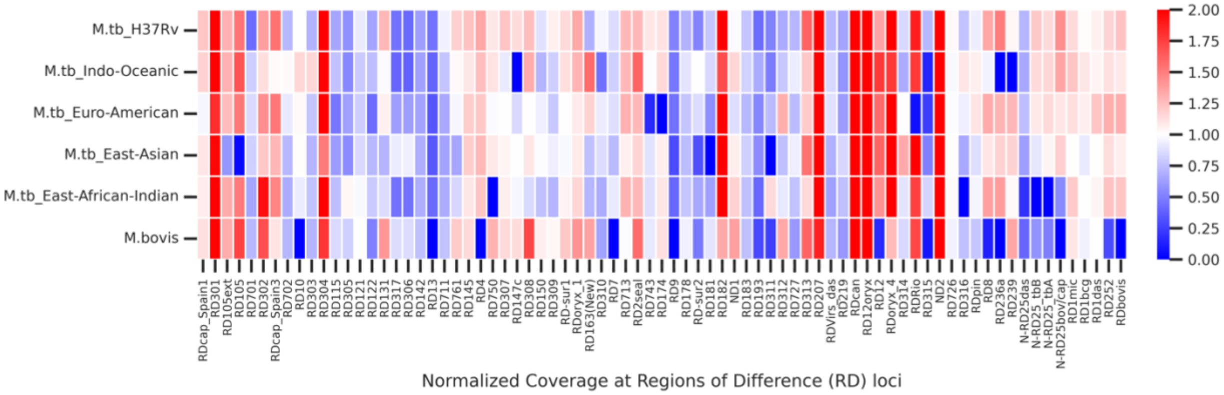

Figure S13: Heatmap of raw normalized coverage from WGE-CNERs data at RD loci for six MTBCs captured with 10,000- (A) and 1000-copy (B) mixtures.
